# Evaluation of a Digital Food and Nutrition Literacy Survey to Support Improvements in Diet Quality

**DOI:** 10.1016/j.cdnut.2026.109526

**Published:** 2026-09-04

**Authors:** Daniela C Avelino, Ock K Chun, Michael J. Puglisi, Ran Xu, Valerie B Duffy

**Affiliations:** 1 Department of Allied Health Sciences, University of Connecticut, Storrs, CT, United States; 2 Department of Nutritional Sciences, University of Connecticut, Storrs, CT, United States

**Keywords:** digital literacy, food and nutrition literacy, diet quality, online grocery shopping, low-income adults

## Abstract

**Background:**

Using digital tools to support healthy food choices and access may counteract the negative effects of living in underresourced communities on diet quality.

**Objectives:**

This study aims to develop and evaluate the psychometric properties of a Digital Food and Nutrition Literacy (DFNL) survey and its association with diet quality for informing future tailored interventions to improve diet quality.

**Methods:**

The DFNL survey was theory-informed by the Information-Motivation-Behavioral Skills model and a multidimensional model for making healthy food choices online. In this cross-sectional study, 196 adults from a low-income, low–food access community in Northeast Connecticut completed an online survey. Principal component analysis was used to identify a DFNL index and components for multivariable linear regression analyses with diet quality, an external variable theoretically expected to be associated with DFNL. Diet quality was assessed using the Short Healthy Eating Index and liking-based Diet Quality Index.

**Results:**

The 10-item DFNL index was internally reliable and explained 53.8% of total variance through 3 interpretable components—online grocery shopping, online nutrition information, and online communication. Associations between DFNL and diet quality varied by DFNL component and diet quality measure; the strongest associations were observed for online nutrition information and online grocery shopping components, particularly with adequacy scores of both diet quality measures.

**Conclusions:**

These preliminary findings support the construct validity of the DFNL index. Assessing DFNL may help identify modifiable behavioral skills in community nutrition interventions that can be tailored to participants’ knowledge gaps and motivation to support diet quality in underresourced populations.

## Introduction

Poor diet quality is a major risk factor for chronic diseases, including type 2 diabetes, cardiovascular disease, obesity, and some cancers [1,2]. Given the central role of diet quality in chronic disease prevention, digital interventions and technology-mediated communication have emerged as promising tools to promote healthier eating behaviors. A systematic review showed that digital nutrition interventions can improve dietary behaviors, including increased fruit and vegetable intake and healthier household eating practices [[3], [4], [5]]. Technology-mediated communication also supports patient-provider engagement, access to care, and broader health equity goals outlined in Healthy People 2030 [6,7]. Social media has become an important source of food and nutrition information; however, although many adults report that online nutrition content encourages healthier choices, many also encounter conflicting dietary messages [8].

Digital literacy may be an important but often overlooked factor influencing healthy eating behaviors, particularly when individuals search for and apply nutrition information online [9,10]. Digital literacy is increasingly recognized as a social determinant of health [11,12], and low digital literacy may limit access to digital health resources, including nutrition education and online diet programs [12]. A scoping review of adult food and nutrition literacy studies found that none evaluated digital literacy skills for navigating online food retail environments [13]. To address this gap, our study integrates 3 related literacy domains: *1*) digital literacy, defined as the ability to find, evaluate, create, and communicate information using digital technologies [14]; *2*) food literacy, which includes the knowledge, motivation, confidence, and skills required to apply dietary guidelines and manage food resources [[15], [16], [17]]; and *3*) nutrition literacy, which involves evaluating and using nutrition-related information, such as nutrition labels, to make informed dietary decisions [17].

Online grocery shopping has become increasingly common, including among households participating in the Supplemental Nutrition Assistance Program (SNAP) [18]. During the COVID-19 pandemic, expansion of the SNAP Online Purchasing Pilot (OPP) increased opportunities for SNAP recipients to purchase groceries online using Electronic Benefits Transfer cards [19]. Online SNAP redemptions increased substantially between 2020 and 2023, reflecting rapid digital adoption [20]. However, low-income adults often experience barriers to online grocery shopping to support healthy eating, including limited nutrition knowledge, lower digital literacy, restricted internet access, added delivery costs, and limited retailer availability [[21], [22], [23]]. These barriers may further widen disparities in diet quality within food environments that are increasingly digitalized.

Nutrition educators serving low-income populations have emphasized the need for education tailored to online grocery shopping and digital food environments [24]. Facilitating the use of digital coupons and online sales tools in SNAP-OPP could enhance accessibility, equity, and affordability for healthier foods [25,26]. As food and nutrition assistance programs, grocery shopping, and health-diet-related resources continue moving online, strong Digital Food and Nutrition Literacy (DFNL) skills are becoming increasingly important [9,13]. However, to our knowledge, no existing tool comprehensively assesses DFNL among low-income adults in the United States.

This study describes the development and evaluation of a survey to assess DFNL that is theory-informed based on the Information-Motivation-Behavioral Skills (IMB) model [27] and a multidimensional literacy model [9,13]. The aims of this study were to: *1*) develop a DFNL survey for adults living in a low-income, low–food access community; *2*) evaluate the underlying structure of survey responses to form a DFNL index; *3*) describe participant DFNL behaviors and motivation to improve DFNL skills; and *4*) examine associations between DFNL scores and diet quality. These findings can inform future community nutrition interventions and digital health strategies aimed at improving diet quality among low-income adults.

## Methods

The DFNL survey’s development and preliminary psychometric evaluation followed a systematic, multistep process to assess face validity, cognitive validity, construct validity testing, and internal reliability. Table 1 outlines each step of the process and methods informed by previous research with low-income communities [28,29]. The study was approved by the UConn Institutional Review Board (Protocol #X22-0013).

**TABLE 1:** Overview of the methods used for the development and validation of the DFNL survey

Step	Type of validity/reliability being assessed	Methods used
1. Concept development	Define the concepts to identify the level of DFNL and integrate with the behavior change theory (IMB model) into an overall conceptual framework	Literature search and expert opinion
2. Face validity	Draft and refine questions to represent the DFNL and IMB conceptual models	Literature search and review by experts in digital literacy, nutrition, and public health
3. Cognitive validity	Assess the clarity and relevance of the online survey for participants.	Pilot testing and think-aloud method with 6 community participants to improve participant understanding of the questions.
4. Construct validity testing	• PCA: assess the underlying structure of a DFNL index. • Hypothesis testing: assess the association between DFNL and diet quality, an external variable theoretically expected to be associated with DFNL.	Testing the survey with 150–200 residents from the target community to determine if the index reflected >1 underlying dimension, and the derived components explained >50% of the total variance. Regression analysis controlling for potential confounding variables.
5. Reliability testing	Internal reliability: assess the internal consistency of the index.	Cronbach’s *α* testing for assessing internal reliability of the DFNL index.

Abbreviations: DFNL, Digital Food and Nutrition Literacy; IMB, Information-Motivation-Behavioral-Skill; PCA, principal component analysis.

### Concept development

A literature search informed the concepts needed to assess the level of DFNL as well as the conceptual framework for the survey that integrated 2 theoretical models to understand individual and modifiable DFNL behaviors related to diet quality and diet-related health (Figure 1). The first integrated theoretical model, the IMB model, posits that an individual would act on a behavior if they had sufficient and accurate knowledge, the motivation or willingness to do the behavior, and the skills and confidence to perform the behavior [27]. The second model identifies concepts of DFNL that can be characterized as ranging from the lowest level, functional, to the higher levels of interactive, communicative, and critical [9,13].

**FIGURE 1:**
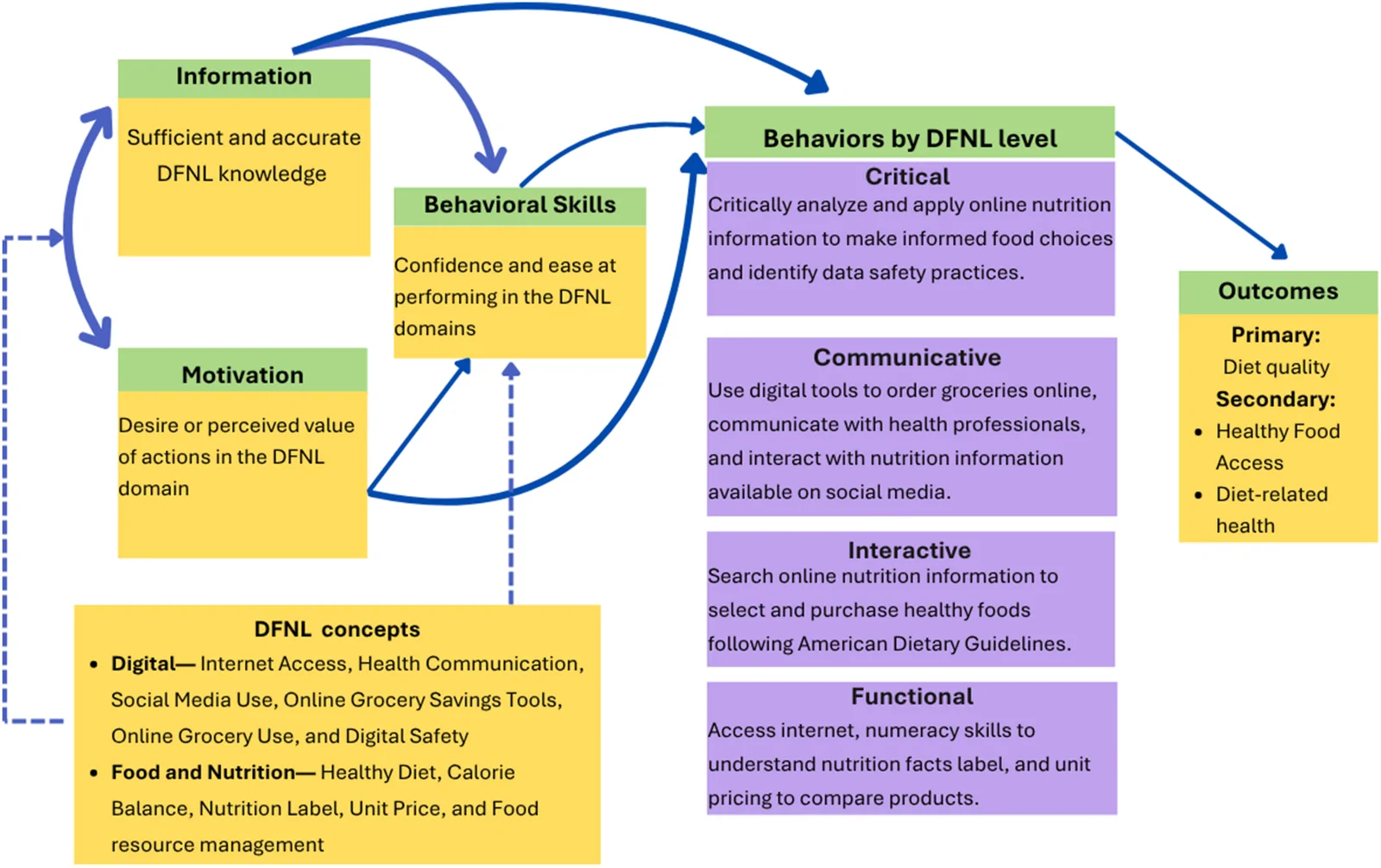
Conceptual framework combining a behavioral theory—the Information, Motivation, and Behavioral Skills [27]—with a multidimensional digital food and nutrition literacy model [9,13] to support diet quality and health. DFNL, Digital Food and Nutrition Literacy.

Bloom’s taxonomy of measurable verbs was used to support the organization and description of survey items [30]. TABLE 2, TABLE 3 describe survey items organized according to the IMB model [27]. Table 2 addresses digital literacy [34,35] and digital food and nutrition environment concepts [25,26]. Table 3 focuses on food and nutrition literacy concepts aligned with previous food and nutrition literacy research [15,17,36] and an assessment tool developed for low-income adults [16]. Survey items for digital literacy behaviors included *1*) access to internet and devices, *2*) use of health communication tools such as e-mail, patient portals, and video calls, *3*) social media use for nutrition information and engagement, *4*) use of online grocery savings tools such as store apps and digital coupons, *5*) participation in online grocery shopping and cost-saving strategies, and *6*) digital safety practices such as recognizing and avoiding online scams. Food and nutrition literacy behaviors included *1*) awareness, motivation, and use of MyPlate for healthy eating, *2*) reading and applying nutrition labels to make dietary choices, *3*) using unit pricing to compare products, and *4*) food resource management strategies such as budgeting, planning, coupon use, and price comparisons. MyPlate was included based on the evidence of healthy eating patterns, historical precedent [37], support of the plate graphic for healthy eating from scholarly organizations (e.g., American Institute for Cancer Research, American Heart Association, and Harvard T.H. Chan School of Public Health) [[38], [39], [40]], and wide use in nutrition education across the United States. In addition, MyPlate has been integrated into digital nutrition education interventions, such as a pilot study that promoted healthier food choices and delivered nutrition education via text messaging [41]. Thus, in the context of DFNL, MyPlate was regarded as a pertinent indicator of healthy eating awareness.

**TABLE 2:** Digital literacy concepts categorized by the IMB model

Digital literacy concepts	IMB construct	Objectives
Internet access	Information	• Identify current internet and device access • Identify reasons for lack of high-speed internet at home • Recall internet affordability programs
	Motivation^1^	• Willingness to learn about high-speed internet at home
	Behavioral skill	• Assess if there is reliable internet connectivity • Assess frequency of internet connectivity issues among those who lack reliable high-speed internet
Health communication	Information	• Recognize tools to contact providers
	Behavioral skill	• Use digital health communication tools
Social media use	Information	• Recognize social media platforms • Identify the types of nutrition-related information searched online
	Motivation^1^	• Interest in private Facebook food/nutrition groups
	Behavioral skill	• Search for food/nutrition information online and social media platforms
Online grocery savings tools	Information	• Recognize ways to receive sales alerts
	Motivation^1^	• Willingness to learn about online sales tools
	Behavioral skill	• Use digital methods to find grocery sales • Use digital coupons
Online grocery use	Information	• Recognize cost-saving delivery subscriptions
	Motivation^1^	• Interest in online grocery shopping • Interest in digital tools to improve online grocery shopping experience
	Behavioral skill	• Online grocery ordering frequency • Use digital tools to improve the online grocery shopping experience • Managing grocery costs
	Confidence	• Perceive the ease of use of digital tools to improve the online grocery shopping experience
Digital safety	Information	• Recognize online scams
	Motivation^1^	• Willingness to learn about online safety

Abbreviation: IBM, Information-Motivation-Behavioral-Skill.

1 Participants were classified as motivated if they reported a score of ≥15 on the liking scale. The scale ranged from −100 to 100, with “love it”/“hate it” corresponding to ±75–100, “really like/dislike it” to ±45–75, “like/dislike it” to ±15–45, and “it is ok” to −15 to 15.

**TABLE 3:** Food and nutrition literacy concepts and assessment items categorized by the IMB model

Food and nutrition literacy concepts	IMB construct	Objectives
Healthy eating	Information	MyPlate • Recognize MyPlate. • Recall a MyPlate recommendation.
	Motivation^1^	MyPlate • Interest in learning about MyPlate. Healthy eating goals • Identify top 3 diet-related health goals. Nutrition topics • Identify preferred learning topics related to eating healthy (e.g., meal planning, recipes).
	Behavioral skill	MyPlate • Use online tools to learn more about and try MyPlate recommendations.
Nutrition label	Information	• Identify barriers to using nutrition labels. • Use nutrition labels to identify healthier products.
	Motivation^1^	• Willingness to learn about reading labels.
	Behavioral skill	• Frequency of reading labels for dietary selection.
		• Frequency of reading labels online.
		• A 6-question health literacy instrument measures whether individuals can use the nutrition facts label to calculate calories per serving, limit intake, deduct nutrients, and apply it to daily intake [31,32].
Unit price	Information	• Recognize unit price while shopping.
	Motivation^1^	• Interest in learning how to use unit prices.
	Behavioral skill	• Compare products using unit price.
Food resource management	Behavioral skill	• The questionnaire measures food resource management through planning behaviors (such as identifying available food items, planning menus, and making a grocery shopping list) and saving behaviors (seeking sales or coupons and comparing prices) [33].

Abbreviation: IBM, Information-Motivation-Behavioral-Skill.

1 Participants were classified as motivated if they reported a score of ≥15 on the facial-labeled hedonic scale. The scale ranged from −100 to 100, with “love it”/ “hate it” corresponding to ±75–100, “really like/dislike it” to ±45–75, “like/dislike it” to ±15–45, and “it is okay” to −15 to 15.

### Face validity

Expert review was conducted to establish face validity. The draft survey was reviewed and refined by experts in digital literacy, nutrition, and public health with experience working with low-income populations and those who speak Spanish. Experts were invited to provide feedback on the DFNL survey via e-mail. The review focused on conceptual clarity, relevance, and alignment with food and nutrition decision making.

### Cognitive validity

Question pretesting was conducted to assess cognitive validity. Only the DFNL survey was included in the pretesting phase, as the purpose was to evaluate the clarity, comprehension, and usability of the DFNL questions; diet quality, demographic, and health-related questionnaires were not pretested. The survey was pilot tested with 6 participants in community settings. Research assistants used 2 techniques: *1*) a think-aloud [42], in which participants verbalized their thought processes while answering questions, and *2*) follow-up probing, in which participants explained how they understood and interpreted selected questions. Feedback from this testing phase led to minor wording revisions. The final version of the tool was designed to be concise, with an average completion time of <30 min.

### Participants and procedures

A cross-sectional study was conducted in a Northeast Connecticut community with limited access to affordable food, as identified by the USDA’s Food Access Research Atlas database [43]. The area has a large Hispanic/Latino population (47%) [44] and high participation in food assistance programs; specifically, 21.76% of residents are enrolled in SNAP and receive benefits [45]. The study sample reflected several key demographics of the target community; however, representativeness cannot be assumed because recruitment used a nonprobability community-based approach. Eligibility criteria included being 19 y of age or older, residing in the targeted zip codes, being able to read and speak English or Spanish, having public health insurance, being the primary grocery shopper for the household, and having an e-mail account to receive the electronic gift card. Because eligibility for public insurance programs (e.g., Medicaid) and SNAP is based on comparable income requirements in the United States, there is significant overlap between these populations. Hence, public health insurance was included as a proxy for lower socioeconomic level. The recruitment goal was 200 low-income participants, including both English and Spanish speakers. We used a recruitment strategy similar to that used in our previous research [46], which resulted in variability in online grocery ordering as a proxy for differences in digital literacy, as well as variability in reported diet quality.

During 2024, participants were recruited from low-income and/or low-access areas where residents commonly gathered for various activities through word-of-mouth and with support from community collaborators. Recruitment locations included nonprofit organizations, community centers, low-income housing, food pantries, schools, and town library. To promote inclusivity and accessibility, bilingual English and Spanish outreach materials, including flyers and posters, were distributed in the community. To participate, potential participants scanned a QR code or entered the website URL printed on the recruitment flyer, which then led to study information and eligibility questions. Only individuals who met the eligibility criteria could proceed to the full survey. Eligible participants first reviewed the electronic informed consent document before deciding whether to participate. Participants were informed that participation was voluntary and that they could withdraw from the study at any time without penalty. Participant flow data (e.g., the number of individuals screened and eligible) was not systematically tracked because recruitment occurred through multiple dissemination channels and survey links. Consequently, these data could not be reported.

In locations with free Wi-Fi, Chromebooks were provided for in-person recruitment. Participants could use study-supplied devices to take the survey or provide their e-mail addresses to receive a survey link. Completely automated public turing test to tell computers and humans apart verification was used to prevent responses from nonhuman sources, and cookie technology was used to reduce duplicate responses. Participants received a $25 electronic gift card. During data management, researchers maintained participant confidentiality by anonymizing or deidentifying responses, assigning unique identifiers, and limiting access to personally identifiable information to authorized personnel.

The conceptual framework guided the design of the survey questions that were responsive to participant behaviors and intended to support future behavior change strategies. For example, when participants responded that they were not engaging in a specific behavior, a tailored follow-up question assessed their motivation to adopt that behavior. The purpose of the follow-up questions produced by survey branching was also to tailor them to participants’ knowledge. Examples of knowledge questions include those about nutrition labels, unit pricing, and MyPlate.

### Measures

In addition to the DFNL survey, participants completed the following questionnaires.

#### Diet quality

Two validated measures assessed dietary quality. The Short Healthy Eating Index (sHEI) Survey is a simple and reliable method to assess diet quality by asking people about daily frequency or usual consumption of different food groups, such as vegetables, fruits, fruit juice, whole grains, refined grains, beans, seafood, and dairy products [47]. Similar to the United States Healthy Eating Index, the sHEI ranges from 0 to 100, comprising contributions of adequacy and moderation scores, with higher sHEI scores representing higher diet quality. For the liking-based Diet Quality Index (DQI), participants rate their likes or dislikes of food and health behavior items as well as physical activities and sedentary behaviors [48] with a sliding scale labeled with 7 faces and verbal descriptors [49,50]. The scale score ranges from 100 to −100, with love it as ±75 to 100, really like ±45 to 75, like ±15 to 45, it is ok 15 to −15, dislike –15 to –45, really dislike –45 to –75, and hate it –75 to –100. The scores for each food group were calculated, and weights were assigned based on the Dietary Guidelines for Americans 2020 [37]. The weighting was as follows: vegetables (+3), fruits (+2), fiber-rich foods (+3), high-fat foods (−3), healthy fats (+2), high-fat proteins (−3), low-fat proteins (+3), sweets (−3), and refined carbohydrates (−3). The liking-based DQI is comprised of contributions of adequacy and moderation scores, with higher scores representing higher diet quality and a maximal range from –278 to 278 (e.g., –278 would be hate it ratings for all adequacy foods and love it for all moderation foods).

#### Demographic and lifestyle characteristics

After completing the DFNL survey, participants provided information on gender, sex assigned at birth, race/ethnicity, birthplace, native language, household size, number of years lived at present address, education, employment, marital status, body size from the figure rating scale [51,52], cigarette smoking status including e-cigarette use, physical activity, self-rated health, self-rated diet quality, history of chronic illnesses, food security [53], and nutrition security status [54]. Participants were also characterized by the number of food assistance programs in which they participated, including government- and nongovernment-funded programs.

#### Construct validity testing

Construct validity was assessed using principal component analysis (PCA) and through hypothesis testing. The sample size was guided by recommendations for PCA, which suggest a minimum of 10 respondents per survey item and/or an overall sample of 200–300 observations [55]. Before conducting PCA, the responses from “check all that apply” questions were summed to capture the extent of digital resources and behaviors (e.g., digital devices available, social media platforms used, digital tools used to communicate with healthcare providers, nutrition-related topics searched online). Zero scores were given to participants who reported “none of the above.” There were no missing datapoints.

We used PCA with Varimax rotation to assess how the observed DFNL items were related and to identify an index comprising a clear set of understandable components. PCA was selected to reduce the dimensionality of the survey items and summarize the total variance in the data while evaluating whether the resulting components aligned with theoretically meaningful DFNL dimensions. Because our objective was data reduction rather than modeling latent constructs, we did not seek to partition common variance from unique and error variance, as in exploratory factor analysis. As a result, we interpret the dimensions as components, not latent factors. The items included in the DFNL survey were assessed for suitability with the Kaiser–Meyer–Olkin test (KMO; optimal value: >0.60) and Bartlett’s test of sphericity (optimal value: *P* < 0.05). Items with factor loadings <0.40 were eliminated. At least 3 items had to be retained by each component. Principal axis factoring (PAF) was conducted as a sensitivity analysis to assess the stability of the domain structure. The consistency of the identified domains was evaluated by comparing the PAF factor structure with the PCA component solution.

To evaluate hypothesis testing, multivariable linear regression analyses were conducted to determine whether the DFNL index and component scores were associated with diet-quality measures. Potential covariates were selected based on bivariate associations with the outcome (*P* ≤0.25), following a predefined statistical criterion. This statistical approach was used to identify potential confounders and was not intended to replace a causal framework, such as directed acyclic graphs, for confounder selection. Age was log transformed before inclusion in the regression models because its distribution was positively skewed. The transformation improved adherence to the assumptions of linear regression.

#### Statistical analyses

SPSS software (version 29.0, SPSS Inc.) was used for statistical analysis with a significance criterion of *P* ≤ 0.05 unless otherwise indicated. Descriptive statistics were used to summarize participants’ demographic, lifestyle, and health-related characteristics. Results are reported as means with SE and frequencies with corresponding percentages, as applicable. Appropriate statistical tests, including *t* tests, analysis of variance, and correlation analyses, were used to assess associations among participant characteristics, DFNL index scores, and diet-quality measures, including sHEI and liking-based DQI.

## Results

### Sociodemographic characteristics and diet quality

Table 4 presents unadjusted comparisons of diet quality scores measured by the sHEI and DQI across sociodemographic and health-related characteristics among the participants (*n* = 196). The sample was predominantly female (65.3%), and nearly half (45.4%) of participants were born in Connecticut; 34.7% were born in another country. Households with children accounted for 52.0% of the sample, and 36.7% of participants reported participating in 2 or more food assistance programs. Approximately half of the participants reported poor or fair self-rated health (46.4%) and poor or fair self-rated diet quality (50.5%). Compared with Connecticut data from the Behavioral Risk Factor Surveillance System 2024, DFNL participants reported poorer overall health than the average Connecticut resident [56]. A higher proportion of DFNL participants rated their health as fair (39.3% compared with 12.3%), whereas fewer reported their health as very good (20.6% compared with 36.6%) or excellent (8.2% compared with 21.8%). Although overweight and obesity prevalence were similar between groups, these findings suggest that DFNL participants may experience a greater overall health burden [56]. Regarding body size grouped into weight status categories, 69.9% were classified as overweight or obese. Most participants reported <150 min of physical activity per week, and 26.5% of the sample were current smokers. Statistically significant differences in sHEI scores were observed by age (*P* = 0.025), gender (*P* < 0.001), birthplace (*P* = 0.013), self-rated health (*P* < 0.001), self-rated diet (*P* < 0.001), and smoking status (*P* = 0.018). Higher sHEI scores were found among participants born outside Connecticut or the United States and those reporting better perceived health or diet quality. Similar trends were seen for liking-based DQI scores, with statistically significant differences by birthplace, self-rated health, self-rated diet, and smoking status.

**TABLE 4:** Unadjusted measures of diet quality by sociodemographic and health characteristics among adults living in a low-income, low–food access community in Northeast Connecticut (*n* = 196)

	Total (*n* = 196) % or M (SD)	sHEI score		Liking-based DQI^1^	
Characteristic		Mean	*P* value	Mean	*P* value
Age			0.025		0.054
19–38	54.6 (107)	50.5		91.4	
≥39	45.4 (89)	47.5		107.1	
Gender			<0.001		0.692
Male	34.7 (68)	45.8		6.2	
Female	65.3 (128)	50.9		11.2	
Race					
White/Caucasian	41.3 (81)	47.5	0.090	4.4	0.741
Latino/Hispanic	45.4 (89)	51.0		13.3	
Black/African American	4.1 (8)	47.9		19.2	
Others^2^	9.2 (18)	47.9		8.5	
Birthplace			0.013		0.016
Connecticut (CT)	45.4 (89)	47.6		4.0	
U.S. outside CT	19.9 (39)	47.9		–1.5	
Another country	34.7 (68)	51.8		22.7	
Household size			0.280		0.993
Adults only	48.0 (94)	48.4		7.7	
Households with children	52.0 (102)	49.8		11.0	
Marital status			0.541		0.390
Married/partner	32.7 (64)	48.5		10.6	
Separated/divorced/widowed	26.0 (51)	50.3		14.8	
Never married	41.3 (81)	48.8		5.1	
Education			0.243		0.904
≤8th grade/some high school	17.3 (34)	51.7		8.6	
H.S. graduate/GED	29.1 (57)	49.4		8.1	
Some college or technical	31.1 (61)	47.8		8.3	
Graduate/professional degree	22.4 (44)	48.4		13.2	
Employment			0.344		0.107
Full-time	23.0 (45)	48.2		7.3	
Part-time employment	20.9 (41)	47.7		6.5	
Unemployed, active seeking	29.1 (57)	50.8		4.9	
Unemployed, not seeking^3^	27.0 (53)	49.2		18.3	
Body size category^4^			0.217		0.721
Normal weight	29.1 (57)	50.2		8.3	
Overweight	44.4 (87)	49.6		11.2	
Obesity	25.5 (50)	47.2		8.2	
Self-rated health status			<0.001		0.003
Poor/fair	46.4 (91)	46.4		–2.0	
Good	36.2 (71)	51.4		16.5	
Very good/excellent	17.3 (34)	51.8		25.2	
Self-rated diet status			<0.001		<0.001
Poor/fair	50.5 (99)	46.7		–3.0	
Good	32.7 (64)	50.7		17.3	
Very good/excellent	16.8 (33)	53.3		31.4	
Food security status^5^			0.158		0.177
Food secure	32.1 (63)	50.0		14.0	
Low food security	35.2 (69)	50.0		11.6	
Very low food security	32.7 (64)	47.3		2.5	
#Drivable vehicles			0.807		0.533
0 vehicles	39.7 (77)	48.9		8.9	
≥1 vehicle	60.3 (117)	49.3		9.9	
Food assistance participation^6^			0.850		0.646
1 program	41.8 (82)	49.6		10.5	
≥2 programs	36.7 (72)	48.9		5.4	
None	21.4 (42)	48.6		14.2	
Cigarette smoking			0.018		<0.001
Current (including e-cigarettes)	26.5 (52)	47.1		–9.1	
Former	18.9 (37)	47.0		20.6	
Never	54.6 (107)	50.8		14.6	
Physical activity/wk			0.754		0.074
<150 min	79.6 (156)	49.0		6.6	
≥150 min	20.4 (40)	49.5		20.6	

Abbreviations: DQI, Diet Quality Index; GED, general educational development.

1 The liking-based DQI was assessed by survey-reported food likes/dislikes and conceptual food groups that were conceptually weighted following the Dietary Guidelines 2020 and averaged [37].

2 Includes Asian/South Asian/Pacific Islander, American Indian or Alaskan Native, or multiracial.

3 Include student, retired, homemaker, disabled, etc.

4 Assessed by Stunkard’s figure rating scale normal [51,52] weight (1–4= Normal weight, 5–6 = Overweight, and 7–9 = Obese) (2 missing data).

5 Assessed by a 6-item food security interview [53].

6 Includes Child and Adult Care Food Program, food pantry, and mobile food trucks.

The sHEI scores indicated that the proportion of participants meeting United States Dietary Guidelines recommendations varied across food groups. Fewer than half met recommendations for vegetables, whole grains, milk, low-fat dairy, and nuts/seeds. In contrast, a greater proportion met recommendations for whole fruit, beans/peas/lentils, and limited intake of sugar-sweetened beverages, although adherence remained inconsistent across dietary components (Supplemental Material 1). The sHEI showed good variability, ranging from 24.5 to 71.1, and was approximately normally distributed, with a mean of 49.1 ± 9.3 [95% confidence interval (CI): 47.8, 50.4]. The liking-based DQI also showed good variability, with a mean of 9.4 ± 41.4 (95% CI: 3.6, 15.3). All food groups averaged within the liking range, with the highest liking for fruit and the lowest for sweets and sugar-sweetened beverages. Exercise liking averaged below all food groups (Supplemental Material 2). The groups comprising the DQI demonstrated very good internal reliability (α = 0.876).

### Digital food and nutrition literacy characteristics

#### Online grocery shopping

Of those who used ≥1 online grocery shopping tool (61.2%, *n* = 120), most checked for online grocery sales each week (63.4%), and 47.5% used digital coupons weekly. Almost half (45.9%) never ordered groceries online, whereas 42.4% ordered at least once a month, including 19.4% who ordered weekly or more. Among those who ordered groceries online (*n* = 106), 39.6% incorrectly responded to the online safety question about unsolicited e-mails or text messages. Most participants (90.3%, *n* = 177) correctly identified the healthier drink using the nutrition facts labels; however, among those who received a follow-up question, 30.0% incorrectly explained why the selected drink was healthier. Among participants who reported using unit price information while shopping (45.9%, *n* = 90), 66.7% correctly identified the better deal based on unit price.

#### Online nutrition information

Most (87.2%) searched for nutrition information online in the last month. Recipes were the most common search (57.7%), followed by healthy eating tips (35.7%) and healthy eating on a budget (34.7%). About 63.2% (*n* = 117) used social media platforms for recipes, nutrition facts, or shopping tips ≥2 to 3 times a week, with some doing so daily. Although most (76.0%, *n* = 149) had heard of MyPlate, fewer (40.3%) knew its recommendation to fill half the plate with fruits and vegetables. In addition, 39.6% checked the MyPlate plan online, and 59.7% attempted to follow its advice.

#### Online communication

Nearly all participants (97.4%, *n* = 191) reported having ≥1 form of internet access, including home Wi-Fi, a mobile phone with cellular service, or a prepaid smartphone. Home Wi-Fi, the most common type of access, was reported by 81.6% (*n* = 160) of respondents. All (*n* = 196) reported having ≥1 device with internet access. Nearly all (94.4%, *n* = 185) used remote communication tools to contact healthcare professionals, most commonly phone calls (73.5%, *n* = 144), followed by e-mail (57.1%, *n* = 112), and online portals (49.5%, *n* = 97). Most participants (70.8%, *n* = 139) used between 2 and 5 digital tools, and nearly all participants (94.4%) used a social media platform. Facebook was the most popular (77.0%, *n* = 151), followed by Facebook Messenger (51.0%, *n* = 100), YouTube (46.4%, *n* = 91), and WhatsApp (44.9%, *n* = 88). More than half (58.2%, *n* = 114) used 3 or more social media platforms.

#### Motivation to work on DFNL

Participants showed interest in learning about and using digital tools and nutrition practices to help them make healthier food choices. Many reported they were willing to use digital resources in different areas. For example, 66.7% (*n* = 24) of those without home Wi-Fi were open to learning how to get high-speed internet, showing they wanted better digital access. On the basis of survey responses and skip logic, a subgroup of 132 participants (67.3%) received a motivation-focused question about online grocery safety. This subgroup included individuals who never ordered groceries online (*n* = 90) and online grocery shoppers who answered the safety question about spam incorrectly (*n* = 42). Of the subgroup, 67 (50.8%) were willing to learn more about online safety. Also, 38.9% (*n* = 35) of people who had never ordered groceries online said they would be willing to try it.

Participants were also motivated to use digital nutrition resources. Of those not using social media platforms for nutrition information, 36.8% (*n* = 21) said they would join private online nutrition groups. In terms of nutrition knowledge, 58.1% (*n* = 79) wanted to learn about MyPlate recommendations, and 52.5% (*n* = 21) of those who did not usually read nutrition labels were willing to learn how to understand them. Likewise, 58.5% (*n* = 62) of people who did not know about unit price information were open to learning how to use it to find better food deals. Detailed item-level results are available in Supplemental Materials 3 and 4.

#### PCA of the DFNL index

The suggested minimum of 10 respondents per survey item was exceeded by the analysis, which comprised 196 participants and 10 indicators (i.e., nearly 19.6 participants per indicator), supporting a sufficient sample size. Bartlett’s test of sphericity (*χ*^2^ = 424.5, *df* = 45, *P* < 0.001) and a KMO value of 0.695 supported that the survey data satisfied the criteria for PCA. Following Kaiser normalization and Varimax rotation, 3 components with eigenvalues >1 were found that explained 53.8% of the variance. The rotated component matrix showed 3 interpretable components, and all items met the prespecified loading threshold. Component 1, 29.2% variance explained, included online grocery-related behaviors (loadings ranged from 0.647 to 0.864). Component 2, 13.7% variance explained, covered online nutrition information seeking (loadings ranged from 0.532 to 0.739). Online communication and nutrition label knowledge comprised component 3, 10.9% variance explained (loadings varied from 0.402 to 0.754). All items met the required thresholds, supporting the validity of these components. Using the same set of indicators in sensitivity analysis, the PAF yielded a broadly similar conceptual structure to that identified by the PCA, although nutrition label knowledge showed a weaker loading than observed in the PCA (Supplemental Material 5). Because the PCA provided a more interpretable component structure with stronger loadings, the PCA solution was retained for the development of the final DFNL index (Figure 2).

**FIGURE 2:**
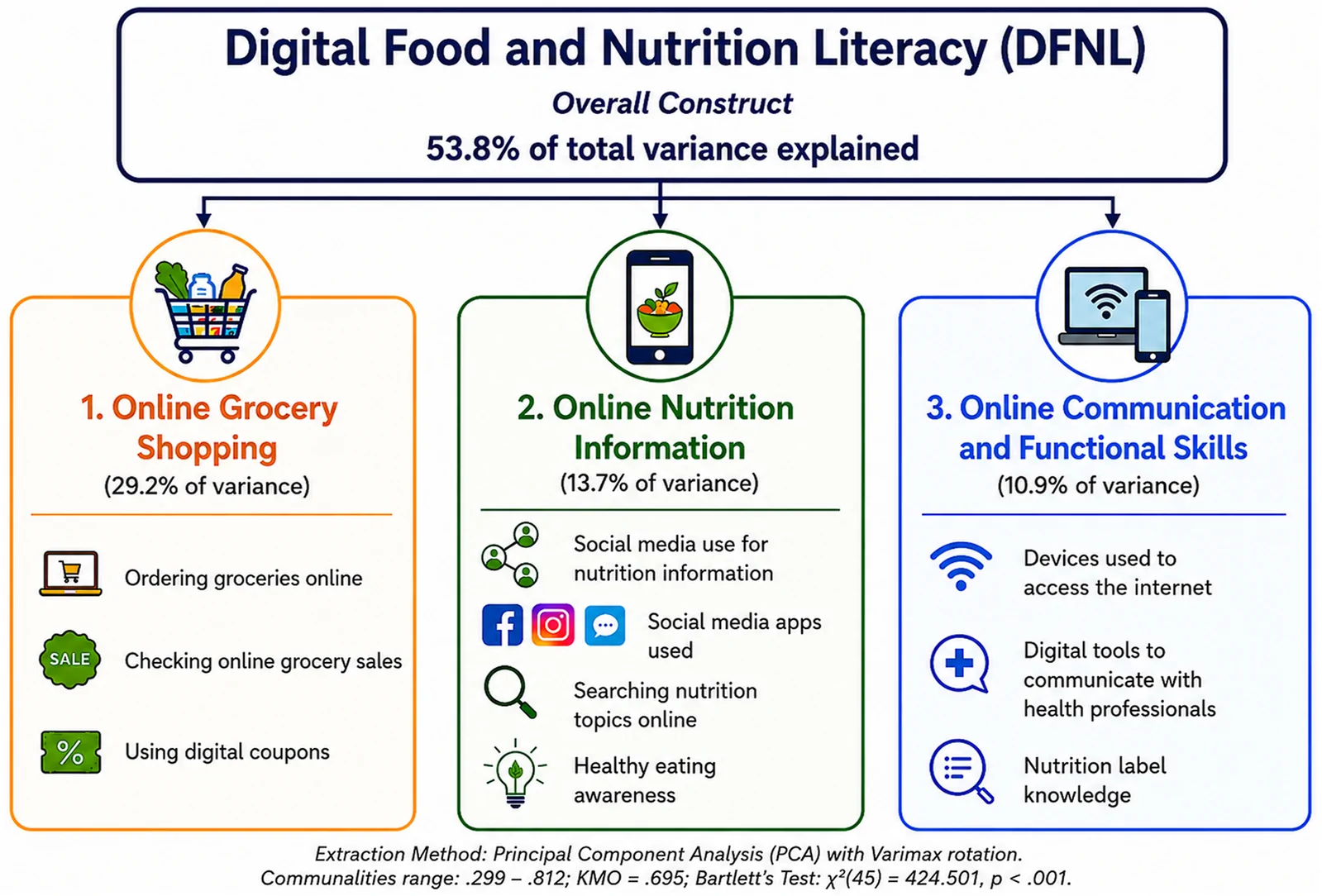
AI-generated figure of the Digital Food and Nutrition Literacy Index with three components—online grocery shopping, online nutrition information, and online communication—together define DFNL. The percent variance explained by each component is shown. DFNL, Digital Food and Nutrition Literacy. The figure was generated with OpenAI ChatGPT (GPT-5.2) in an iterative process to create and refine the figure to include statistical analysis findings, component labels, and component loadings (OpenAI, 2026).

The DFNL index was developed using a standardized scoring approach in which each indicator contributed a maximum of 7 points to the overall index score (Table 5). For indicators based on the sum of related responses (e.g., number of digital devices used, number of digital tools used to communicate with healthcare providers, number of nutrition-related topics searched online, and number of social media platforms used), the maximum score of 7 points was distributed proportionally across the possible responses. For behavior and frequency-based questions, response categories were assigned increasing scores, with higher frequencies or behaviors consistent with greater DFNL receiving higher scores, up to a maximum of 7 points. The scored items and indicators were then combined into the DFNL index score.

**TABLE 5:** DFNL components with questions, scoring, and descriptive findings among adults living in a low-income, low–food access community in Northeast Connecticut (*n* = 196)

	Survey item	Item range	Number of items	Total possible range	Mean ± SD
Component 1—Online grocery shopping (29.2% variance)					
Frequency of checking online grocery sales	How often do you go online to check grocery sales? 0 = Never, 1.75 = Every 3 mo, 3.5 = Once or twice a month, 5.25 = Once a week, 7 = > once a week	0–7	1	0–7	3.0 ± 2.8
Frequency of using digital coupons	How often do you use digital coupons to buy groceries? 0 = Never, 1.75 = Every 3 mo, 3.5 = Once or twice a month, 5.25 = Once a week, 7 = > once a week	0–7	1	0–7	2.3 ± 2.7
Frequency of ordering groceries online	How often do you order groceries online? 0 = Never, 1.75 = Every 3 mo, 3.5 = Once or twice a month, 5.25 = Once a week, 7 = > once a week	0–7	1	0–7	2.2 ± 2.4
Component 2—Online nutrition information (13.7% variance)					
Frequency of using social media for nutrition	How often did you go on social media for recipes, nutrition information, or tips on food shopping in the last month? 0 = Never, 1.4 = Once a month, 2.8 = 2–3 times a month, 4.2 = 2–3 times a week, 5.6 = Once a day, 7 = Many times a day	0–7	1	0–7	3.7 ± 2.3
Number of nutrition information topics searched online	What nutrition information did you search online in the last month? 0 = None 1 = Grocery shopping tips, 1 = Healthy eating on a budget, 1 = Maintain a healthy eating lifestyle, 1 = Meal planning, 1 = Recipes, 1 = Time-saving meal prep, 1 = Vegetarian meals	0–7	1	0–7	2.2 ± 1.8
Number of social media apps used	Sometimes, people can connect with others online through social media or online chat groups. Check the social media or online chat groups you have used in the last month 0 = None 1 = Facebook, 1 = Instagram, 1 = Twitter, 1 = TikTok 1 = YouTube, 1 = WhatsApp, 1 = Facebook Messenger	0–7	1	0–7	2.9 ± 1.7
Healthy eating awareness	Have you heard of MyPlate? 0 = No 7 = Yes	0–7	1	0–7	5.3 ± 3.0
Component 3—Online communication and functional skills (10.9% variance)					
Number of devices to access the internet	Please indicate what device(s) you use to access the internet. Check all that apply 0 = Devices from public spaces 1.75 = Prepaid smartphone 1.75 = Smartphone (with service/cellular data), 1.75 = Tablet, 1.75 = Laptop	0–7	1	0–7	2.8 ± 1.4
Number of digital tools to communicate with health professionals	Do you use any of the following digital tools to communicate with health professionals? Please select all that apply 0 = None of the above, 1.4 = Phone call, 1.4 = E-mail, 1.4 = Online portals, 1.4 = Video conferencing, 1.4 = Text messaging	0–7	1	0–7	3.6 ± 2.1
Nutrition label knowledge	Please compare the nutrition labels for 2 drinks. Which drink do you think is healthier? 0 = Incorrect 7 = Correct	0–7	1	0–7	6.3 ± 2.1
Total DFNL index	(α = 0.703)	4.2–68.3	10	0–70	34.4.2 ± 11.9

Abbreviation: DFNL, Digital Food and Nutrition Literacy.

The final index included 10 items across 3 components: online grocery shopping (3 items), online nutrition information (4 items), and online communication (3 items). Participants reported moderate use of online tools for nutrition information (e.g., social media platforms: M = 3.7 ± 2.3) and online grocery shopping (e.g., digital coupons: M = 2.3 ± 2.7). Because the PCA identified 3 components, Cronbach's α was interpreted as an index-level measure of internal consistency. The combined DFNL index was used in subsequent analyses because it demonstrated acceptable internal reliability (Cronbach’s *α* = 0.703), a wide range (4.1–68.2), and a normal distribution based on visual inspection. Higher scores indicated greater engagement with digital food, nutrition, and communication behaviors.

#### Associations between DFNL and diet quality

The DFNL index scores showed a significant association with the sHEI only before adjustment for the demographic and health-related covariates included in the multivariable model (*β* = 0.112, *P* = 0.044) (Table 6). Among the DFNL components, increased engagement with online nutrition information was associated with higher sHEI in unadjusted (*β* = 0.396, *P* < 0.001) and adjusted models (*β* = 0.309, *P* = 0.007). The liking-based DQI showed no significant association between the DFNL index scores before and after adjustment. Online grocery shopping was positively associated with DQI after adjustment (*β* = 1.009, *P* = 0.016). Online communication did not demonstrate significant associations with the sHEI or the liking-based DQI.

**TABLE 6:** Associations of the DFNL index and components with the sHEI and liking-based DQI scores among adults living in a low-income, low–food access community in Northeast Connecticut (*n* = 196)

sHEI score							Liking-based DQI score					
		*β*^1^	Standardized *β*	*t*	95% CI	*P* value		*β*^1^	Standardized *β*	*t*	95% CI	*P* value
DFNL index	Crude linear models	0.112	0.144	2.026	0.003, 0.221	0.044	Crude linear models	0.228	0.07	0.97	–0.236, 0.692	0.333
	Adjusted linear model	0.094	0.121	1.699	–0.015, 0.203	0.091	Adjusted linear model	0.426	0.13	1.79	–0.045, 0.897	0.076
Online grocery shopping	Crude linear models	0.136	0.095	1.329	–0.066, 0.337	0.186	Crude linear models	0.752	0.125	1.75	–0.095, 1.599	0.081
	Adjusted linear model	0.183	0.129	1.92	–0.005, 0.370	0.056	Adjusted linear model	1.009	0.168	2.43	0.189, 1.829	0.016
Online nutrition information	Crude linear models	0.396	0.253	3.649	0.182, 0.611	<0.001	Crude linear models	–0.041	–0.006	–0.09	–0.975, 0.892	0.93
	Adjusted linear model	0.309	0.197	2.713	0.084, 0.533	0.007	Adjusted linear model	0.211	0.032	0.42	–0.777, 1.198	0.674
Online communication	Crude linear models	–0.28	–0.11	–1.538	–0.629, 0.078	0.126	Crude linear models	0.156	0.015	0.21	–1.339, 1.652	0.837
	Adjusted linear model	–0.26	–0.105	–1.545	–0.600, 0.073	0.124	Adjusted linear model	0.468	0.045	0.63	–0.988, 1.925	0.527

Abbreviations: DFNL, Digital Food and Nutrition Literacy; sHEI, Short Healthy Eating Index.

Model adjusted for age (log transformed), gender (male/female), birthplace [1 = Connecticut (CT), 2 = United States outside CT, 3 = another country], education (1 = ≤8th grade/some high school, 2 = high school graduate/general educational development, 3 = some college or technical, 4 = graduate/professional degree), employment (0 = unemployed, not seeking, 1 = unemployed, active seeking, 2 = part-time employment, 3 = full-time), food security status (1 = food secure, 2 = low food security, 3 = very low food security), perceived health (poor/fair, good, very good/excellent), smoking status (1 = current and former, 0 = never), and physical activity per week (<150/≥150 min).

1 *β*: unstandardized coefficient.

Analysis of the sHEI adequacy and moderation scores (Table 7) indicated that the DFNL index scores were associated with adequacy before and after adjustment (*β* = 0.123, *P* = 0.009; *β* = 0.101, *P* = 0.034) but not with the moderation scores. Online grocery shopping was positively associated with sHEI adequacy scores after adjustment (*β* = 0.167, *P* = 0.045). Online nutrition information consistently demonstrated a positive association with adequacy across all models (*β* = 0.431, *P* < 0.001; *β* = 0.342, *P* < 0.001). Greater online communication was associated with lower sHEI in both models (*β* = –0.31, *P* = 0.04; *β* = –0.31, *P* =0.035).

**TABLE 7:** Associations of the DFNL index and components with the sHEI adequacy and moderation scores among adults living in a low-income, low–food access community in Northeast Connecticut (*n* = 196)

sHEI adequacy score							sHEI moderation score					
		*β*^1^	Standardized *β*	*t*	95% CI	*P* value		*β*^1^	Standardized *β*	*t*	95% CI	*P* value
DFNL index	Crude linear models	0.123	0.186	2.642	0.031, 0.214	0.009	Crude linear models	–0.011	–0.022	–0.3	–0.080, 0.059	0.765
	Adjusted linear model	0.101	0.153	2.136	0.008, 0.194	0.034	Adjusted linear model	–0.007	–0.014	–0.181	–0.080, 0.066	0.856
Online grocery shopping	Crude linear models	0.154	0.128	1.794	–0.015, 0.324	0.074	Crude linear models	–0.019	–0.021	–0.288	–0.146, 0.109	0.773
	Adjusted linear model	0.167	0.138	2.018	0.004, 0.330	0.045	Adjusted linear model	–0.012	–0.013	–0.179	–0.139, 0.116	0.858
Online nutrition information	Crude linear models	0.431	0.326	4.797	0.254, 0.608	<0.001	Crude linear models	–0.034	–0.035	–0.483	–0.174, 0.106	0.63
	Adjusted linear model	0.342	0.258	3.558	0.152, 0.531	<0.001	Adjusted linear model	–0.033	–0.034	–0.432	–0.185, 0.119	0.667
Online communication	Crude linear models	–0.31	–0.147	–2.066	–0.609, –0.014	0.04	Crude linear models	0.036	0.023	0.316	–0.189, 0.260	0.753
	Adjusted linear model	–0.31	–0.145	–2.123	–0.595, –0.022	0.035	Adjusted linear model	0.045	0.028	0.394	–0.180, 0.270	0.694

Abbreviations: DFNL, Digital Food and Nutrition Literacy; sHEI, Short Healthy Eating Index.

Model adjusted for age (log transformed), gender (male/female), birthplace [1 = Connecticut (CT), 2 = United States outside CT, 3 = another country], education (1 = ≤8th grade/some high school, 2 = high school graduate/general educational development, 3 = some college or technical, 4 = graduate/professional degree), employment (0 = unemployed, not seeking, 1 = unemployed, active seeking, 2 = part-time employment, 3 = full-time), food security status (1 = food secure, 2 = low food security, 3 = very low food security), perceived health (poor/fair, good, very good/excellent), smoking status (1 = current and former, 0 = never), and physical activity per week (<150/≥150 min).

1 *β*: Unstandardized coefficient.

Further analyses (Table 8) demonstrated consistent positive associations with liking-based adequacy but not with the moderation scores in adjusted models. The DFNL index and 2 component scores were positively associated with adequacy in unadjusted models, and these significant associations persisted after adjustment for the DFNL index scores (*β* = 0.477, *P* = 0.001; *β* = 0.542, *P* < 0.001), online grocery shopping (*β* = 0.79, *P* = 0.005; *β* = 0.868, *P* = 0.003), and online nutrition information (*β* = 0.689, *P* = 0.023; *β* = 0.714, *P* = 0.038).

**TABLE 8:** Associations of total DFNL index and components with liking-based DQI adequacy and moderation scores among adults living in a low-income, low–food access community in Northeast Connecticut (*n* = 196)

Liking-based DQI adequacy score							Liking-based DQI moderation score					
		*β*^1^	Standardized *β*	*t*	95% CI	*P* value		*β*^1^	Standardized *β*	*t*	95% CI	*P* value
DFNL index	Crude linear models	0.477	0.228	3.22	0.185, 0.769	0.001	Crude linear models	0.4	0.151	2.11	0.025, 0.77	0.037
	Adjusted linear model	0.542	0.252	3.36	0.224, 0.860	<0.001	Adjusted linear model	0.37	0.139	1.81	–0.033, 0.771	0.071
Online grocery shopping	Crude linear models	0.79	0.204	2.87	0.248, 1.331	0.005	Crude linear models	0.51	0.104	1.45	–0.185, 1.204	0.149
	Adjusted linear model	0.868	0.219	3.04	0.305, 1.431	0.003	Adjusted linear model	0.39	0.079	1.08	–0.324, 1.099	0.284
Online nutrition information	Crude linear models	0.689	0.164	2.29	0.096, 1.282	0.023	Crude linear models	0.86	0.161	2.26	0.108, 1.605	0.025
	Adjusted linear model	0.714	0.166	2.09	0.041, 1.386	0.038	Adjusted linear model	0.78	0.147	1.85	–0.052, 1.615	0.066
Online communication	Crude linear models	0.791	0.115	1.6	–0.183, 1.766	0.111	Crude linear models	0.88	0.101	1.41	–0.354, 2.107	0.162
	Adjusted linear model	0.953	0.136	1.84	–0.071, 1.976	0.068	Adjusted linear model	0.62	0.072	0.96	–0.651, 1.896	0.336

Abbreviations: DFNL, Digital Food and Nutrition Literacy; DQI, Diet Quality Index.

Model adjusted for age (log transformed), gender (male/female), birthplace [1 = Connecticut (CT), 2 = United States outside CT, 3 = another country], education (1 = ≤8th grade/some high school, 2 = high school graduate/general educational development, 3 = some college or technical, 4 = graduate/professional degree), employment (0 = unemployed, not seeking, 1 = unemployed, active seeking, 2 = part-time employment, 3 = full-time), food security status (1 = food secure, 2 = low food security, 3 = very low food security), perceived health (poor/fair, good, very good/excellent), smoking status (1 = current and former, 0 = never), and physical activity per week (<150/≥150 min).

1 *β*: unstandardized coefficient.

## Discussion

This study aimed to develop and test a conceptually and theoretically guided survey to measure DFNL and to examine its association with diet quality in adults. Participants were recruited from a community in Northeast Connecticut that experiences challenges with food access, has a large Hispanic/Latino population, and has high participation in food assistance programs. Participants had a broad range of scores on the DFNL items, components, and index, suggesting that the survey can capture differences in DFNL-related behaviors. The 10 items formed a DFNL index with scores that demonstrated acceptable internal reliability based on internal consistency across items and preliminary construct validity from the emergence of interpretable components. The DFNL index scores, in comparison to 2 measures of diet quality, were associated with healthier diet patterns. Higher literacy was associated with higher diet quality from a food frequency-based measure (sHEI) and higher adequacy scores from a frequency and a liking-based measure. The DFNL index components captured literacy behaviors related to online grocery shopping, online nutrition information seeking, and online communication.

Associations with diet quality varied by DFNL component and dietary outcome. Online grocery shopping demonstrated consistent positive associations with both liking-based diet quality and the sHEI adequacy score. Specifically, increased online grocery shopping among SNAP households, as shown in a recent quasi-experimental study, corresponded with more healthful food-at-home purchases [57]. These findings suggest that online grocery shopping behaviors may represent an important component for interventions to support healthier food choices among low-income adults. However, barriers such as cost, delivery options, and limited technology can hinder access and motivation to order groceries online [26]. Evidence from the COVID-19 period suggests that continued adoption of online grocery shopping is strongly influenced by prior experience and positive attitudes toward technology—such as perceived convenience, efficiency, and ease of use—highlighting the role of motivational and skill-based factors [58]. These findings emphasize the need for skills beyond mere access to information. As online grocery shopping expands and new digital tools are introduced, there is a chance to create interventions—such as improved nutrition labels [59] and personalized suggestions—that align with user preferences, build practical skills, and encourage healthier eating [60,61]. Notably, our tailored questions to participants who are not engaging in some DFNL-related behaviors revealed moderate to high interest in understanding healthy eating recommendations, protecting personal data during online grocery shopping, and using unit prices to make cost-effective food choices.

In addition, individuals who reported greater online nutrition information seeking through social media platforms and healthy eating-related behaviors had higher dietary quality scores, supporting the potential role of digital tools in helping individuals identify, interpret, and apply nutrition information to food choices. Social media platforms can serve as digitally mediated sources of information about food, nutrition, and health [62]. In a 2023 survey by the International Food and Information Council, 60% of United States adults who saw food-related content on social media platforms said it encouraged them to make at least somewhat healthier choices [8]. In our study, almost two-thirds of participants reported using social media platforms several times a week to find nutrition-related information (e.g., recipes and healthy eating on a budget). Consistent with our findings, a previous cross-sectional study found that 69% of people in food assistance programs used social media platforms and were interested in nutrition information through these platforms [63]. At the same time, the quality and credibility of online nutrition information vary considerably, highlighting the importance of equipping individuals with the skills needed to critically evaluate and apply nutrition information encountered in digital environments [62].

Digital access and communication resources are important prerequisites for locating, interpreting, and using nutrition information in digital environments [64]. Nevertheless, some overlap with general digital literacy is expected. A systematic review found that individuals with greater digital health literacy feel more confident in evaluating online information, using digital health resources, and participating safely in online health activities [65]. These findings support the need for targeted education for groups with lower digital literacy skills [65]. However, the online communication component showed a different pattern. Higher scores of this component were associated with lower sHEI adequacy scores, and there was no association with the liking-based DQI measure. This was surprising, especially because the component includes a nutrition label knowledge item. If people lack the skills to judge nutrition content, online engagement may not lead to healthier choices and might even cause confusion about healthy eating. Because this study did not include a validated measure of general digital literacy, discriminant validity could not be formally evaluated. Future studies should compare the DFNL index scores with established digital literacy instruments to determine the extent to which it captures nutrition-specific competencies beyond general digital literacy.

From a community nutrition intervention perspective, the DFNL survey may help identify specific modifiable literacy skills that can be targeted through tailored education and support programs. For example, individuals who score lower on online grocery shopping skills could benefit from learning how to use digital coupons, shop with SNAP online, compare unit prices, read nutrition labels on grocery websites, and find ways to reduce delivery costs and fees. Those with lower scores in finding nutrition information online may need help with understanding healthy eating guidelines, spotting reliable nutrition sources, evaluating content on social media platforms, and eating well on a budget. These ideas align well with programs such as the Expanded Food and Nutrition Education Program, food pantry education, and community health promotion, where online delivery has become an important option alongside traditional in-person programming.

This study has some limitations. The sample reflected several key characteristics of the intended population; however, representativeness cannot be assumed because recruitment used a nonprobability community-based approach. There also may be sample bias because more than half of the participants had at least some college education. The comparatively lower liking scores for sweets and sugar-sweetened beverages are consistent with half the sample meeting the Dietary Guidelines for sugar-sweetened beverages (Supplemental Materials 1 and 2). Because the survey was online, adults with limited access to technology or lower digital skills may not have participated. This means the study sample may not fully represent those with lower digital access and literacy, which could limit how well the index score is evaluated across all levels. Given the cross-sectional nature of this study, causal inferences could not be assessed. This study reached a high-need population living in a low-food-access community with a high health burden, as well as captured important differences in DFNL. The DFNL index scores provide a novel measure of digital literacy in food and nutrition contexts and account for multiple demographic and behavioral factors, strengthening the robustness of the findings.

This study serves as the initial exploratory validation of the DFNL instrument. We developed and tested a DFNL survey, providing early evidence of its construct validity through PCA and testing of its association with diet-quality measures. Because the 3-component structure was derived using an exploratory, data-driven approach, including component rotation and item retention based partly on loading criteria, the proposed structure should be considered preliminary. DFNL is important for understanding how individuals make food choices in today’s digital environment. In practice, this tool supports the skills individuals need for online food shopping, finding nutrition information online, and communicating about health digitally. These findings support the need to develop nutrition education and public health programs for digital settings. A necessary next step is to examine the latent structure of the DFNL instrument using common-factor exploratory factor analysis and reproducibility of this structure using confirmatory factor analysis in an independent sample. Future studies should also assess test–retest reliability to determine how stable DFNL scores are over time. Researchers should use survey methods that can reach adults with less digital access and lower digital literacy to evaluate the performance of the DFNL index score across a broader range of digital literacy levels. From a policy perspective, the DFNL framework supports the creation of data-driven digital health and nutrition programs. More studies are needed to determine whether improving DFNL leads to better dietary behaviors and diet quality.

## Author contributions

The authors’ responsibilities were as follows – DCA, VBD, OKC: contributed to conceptualization and methodology; DCA, VBD, OKC, RX: contributed to formal analysis; DCA: contributed to investigation; OKC, VBD: contributed to resources; OKC, VBD, DCA: contributed to data curation; DCA, VBD: contributed to writing—original draft preparation and visualization; VBD, OKC: contributed to supervision; OKC: contributed to project administration; OKC, VBD, MP: contributed to funding acquisition; and all authors: wrote, reviewed, edited, read, and agreed to the published version of the manuscript.

## Ethical approval

This study was conducted in accordance with the guidelines outlined in the Declaration of Helsinki, and all procedures involving research study participants were approved by the Institutional Review Board at the University of Connecticut. Electronic informed consent was obtained from all participants.

## Data availability

The data described in this manuscript, along with the codebook, are available upon request, subject to application to and approval by the corresponding author.

## Declaration of Generative AI and AI-assisted technologies in the writing process

During the preparation of this work, the authors use OpenAI ChatGPT (GPT-5.2) in order to create and refine Figure 2. After using OpenAI ChatGPT, the authors reviewed and edited the content as needed and take full responsibility for the content of this figure.

## Funding

This research was funded by the University of Connecticut Department of Nutritional Sciences Pilot Fund and the USDA-NIFA Agriculture and Food Research Initiative Grant (Program Code A1344) to OKC (grant no. 2023-68015-39601). The funders had no role in the design of the study; in the collection, analyses, or interpretation of data; in the writing of the manuscript; or in the decision to publish the results.

## Conflict of interest

The authors report no conflicts of interest. The content of this paper is solely the responsibility of the authors and does not necessarily represent the official views or policy of USDA-NIFA.
