# Supplementary material for: Evaluation of a Digital Food and Nutrition Literacy Survey to Support Improvements in Diet Quality
Source: Curr Dev Nutr. 2026 Sep 4;10(10):109526. doi: 10.1016/j.cdnut.2026.109526 (PMC13634112; doi:10.1016/j.cdnut.2026.109526)
Supplement: Supplementary file 1 [file mmc1.docx]

**Supplementary Material 1**

Food groups and components of the Short Healthy Eating Index Survey (sHEI) survey (1) that is used to calculate the sHEI score

| **Dietary Component** | **Range** | **Mean** | **Std. Deviation** |
| --- | --- | --- | --- |
| Total Fruits | 0 to 5 | 2.37 | 2.50 |
| Whole Fruit | 0 to 5 | 3.78 | 1.80 |
| Total Vegetables | 0 to 5 | 2.71 | 0.74 |
| Greens and Beans | 0 to 5 | 2.91 | 2.47 |
| Whole Grains | 0 to 10 | 3.89 | 2.17 |
| Dairy | 0 to 10 | 4.35 | 1.29 |
| Total Protein Foods | 0 to 5 | 4.76 | 0.37 |
| Seafood and Plant Protein | 0 to 5 | 2.01 | 1.45 |
| Fatty Acid Ratio | 0 to 10 | 4.47 | 1.27 |
| Refined Grains | 0 to 10 | 4.68 | 3.03 |
| Sodium | 0 to 10 | 4.25 | 1.92 |
| Added Sugars | 0 to 10 | 5.84 | 3.27 |
| Saturated Fats Component | 0 to 10 | 3.11 | 1.48 |
| Total Diet Quality Score | 0 to 100 | 49.12 | 9.25 |

Frequency of participants (n=196) who meet the U.S. Dietary Guidelines (2) food group or component recommendations*.

|  | **Fruit, any kind** | **Whole Fruit** | **Vegetable, any kind** | **Beans, Peas, Lentils** | **Whole Grains** | **Milk, any type** | **Low fat Dairy** | **Seafood** | **Nuts/**  **seeds** | **Sugar-sweetened beverages** |
| --- | --- | --- | --- | --- | --- | --- | --- | --- | --- | --- |
| **Recommendations** | 2 cups/d | 1 cup/d | 2.5 cups/d | 1.5 cups/ wk | 3 oz/d | 3  cups/d | 3  cups/d | 8 oz/wk | 5 oz/wk | ≤12 oz/d |
| **Meeting Dietary Guidelines** | **70 (35.7%)** | **126**  **(64.3%)** | **59**  **(30.6%)** | **114 (58.7%)** | **50**  **(26%)** | **40**  **(20.4%)** | **28 (14.7%)** | **84 (44.4%)** | **56 (29%)** | **106**  **(54.6%)** |

* Recommendations including daily fruit or 100% fruit juice, whole fruit, vegetable, beans, whole grains, milk any type, low fat dairy, limited consumption of sugary drinks.

**Supplementary Material 2**

Average reported liking/disliking of foods and activities (± standard error of the mean) ranked most to least in adults (n = 196) from a low-income, low food access community.

Food groups in the Liking Survey (3) with mean and standard deviation

| **Food Group** | **Mean** | **Std. Deviation** |
| --- | --- | --- |
| Fruits (Bananas, Orange Juice, Pineapple) | 51.98 | 38.50 |
| Healthy Fats (Nuts, Olive Oil) | 40.82 | 38.38 |
| High Fat Protein (Cheddar Cheese, Whole Milk, Pizza, Beef Steak, Bacon, Fried Chicken) | 37.83 | 37.09 |
| Refined Carbohydrates (White Rice, Rolls, Pasta) | 36.98 | 36.41 |
| Vegetables (Broccoli, Carrots, Cabbage, Tomato) | 32.81 | 40.10 |
| Fiber (Whole wheat bread, Beans, Tortilla, Oatmeal) | 29.80 | 40.44 |
| Low-Fat Protein (Tuna, Baked Chicken, Pork Chops) | 24.77 | 38.16 |
| High Fat (Salty Snacks, French Fries, Mayo, Fast Food) | 23.67 | 38.57 |
| Sweet (Ice Cream, Cookies, Soda, Coffee Drinks) | 22.52 | 42.31 |
| Liking-based Diet Quality Index | 9.42 | 41.41 |

**Supplementary Material 3**

Digital Literacy concepts categorized by the Information-Motivation-Behavioral Skills (IMB) Model

| **Concepts** | **IMB Construct** | **Objectives** | **Survey Items** | **% (n)** |
| --- | --- | --- | --- | --- |
| **Internet Access** | Information | - Identify current internet and device access - Identify reasons for lack of high-speed internet at home - Recall internet affordability programs | Internet Access (n=196)  Wi-Fi at home  Phone with service/cellular data  Prepaid Smartphone  Public spaces  Lack internet access  Device Access (n=196)  Phone with aservice/cellular data  Prepaid Smartphone  Tablet  Laptop  Devices from public spaces  Tailored to no home Wi-Fi access through a fixed internet connection  Barriers to high-speed (n=36, 18.4%)  Service is too pricey  No need for home internet  Internet Affordability (n=196)  No  Yes  Don’t Know or Like to Know | 81.6 (160)  30.6 (60)  9.7 (19)  9.7 (19)  2.6 (5)  89.8 (176)  12.2 (24)  24.5 (48)  36.7 (72)  7.7 (15)  69.4 (25)  22.2 (8)  60.7 (119) 26.5 (52)  12.8 (25) |
|  | Motivation* | - Willingness to learn about high-speed internet at home | Tailored to no home Wi-Fi access through a fixed internet connection (n=36, 18.4%) | 66.7 (24) willing to learn* |
|  | Behavioral Skill | - Assess if there is reliable internet connectivity - Assess frequency of internet connectivity issues among those who lack reliable high- speed internet | Reliable Internet Connectivity (n=196)  No  I'm not sure  Sometimes  Yes, without any issues  Tailored to those with  Internet Connectivity Issues (n=81, 41.3%)  2-3 times a month  2-3 times a week  Once a day  Many times a day | 5.1 (10)  5.6 (11)  30.6 (60)  58.7 (115)  17.3 (14)  37.0 (30)  17.3 (14)  28.4 (23) |
| **Health Communication** | Information | - Recognize tools to contact providers | Select all that apply (n=196)  Email  Online Portals  Phone Calls  Video Calls  Text | 57.1 (112)  49.5 (97)  73.5 (144)  36.7 (72)  40.8 (80) |
|  | Behavioral Skill | - Use digital health communication tools | How often (n=196)  Never  Every six months or once a year  Every three months  Once or twice a month  Once a week  More than once a week | 12.2 (24)  11.7 (23)  16.3 (32)  31.1 (61)  8.2 (16)  20.4 (40) |
| **Social Media Use** | Information | - Recognize social media platforms - Identify the types of nutrition-related information searched online | Select all that apply (n=196)  Facebook  Instagram  Twitter  TikTok  YouTube  WhatsApp  Snapchat  Pinterest  Facebook Message  Group text  Select all that apply (n=196)  Recipes  Maintain a healthy eating lifestyle  Healthy eating on a budget  Grocery shopping tips Meal planning  Time-saving meal prep  Dining out/take out  Vegetarian meals | 77.0 (151)  39.8 (78)  5.1 (10)  29.1 (57)  46.4 (91)  44.9 (88)  20.9 (41)  7.7 (15)  51.0 (100)  26.0 (51)  57.7 (113)  35.7 (70)  34.7 (68)  32.7 (64)  31.6 (62)  27.6 (54)  13.8 (27)  9.7 (19) |
|  | Motivation* | - Interest in private Facebook food/nutrition groups | Tailored to no Facebook users or no use of social media for nutrition information (n=57, 29.1%) | 36.8 (21)  willing to join |
|  | Behavioral Skill | - Search for food/nutrition information online and social media platforms | Tailored to those who have a social media account  How often (n=196)  Never  Once a month  2-3 times a month  2-3 times a week  Once a day  Many times a day | 15.8 (31)  11.2 (22)  13.3 (26)  30.6 (60)  11.2 (22)  17.9 (35) |
| **Online Grocery Savings Tools** | Information | - Recognize ways to receive sales alerts | Select all that apply (n= 196)  At the store  By Email  By Text  Grocery store fliers by mailbox  Grocery store ads on social media Searching online  Store App | 60.7 (119)  31.1 (61)  6.6 (13)  33.2 (65)  19.9 (39)  15.3 (30)  27.6 (54) |
|  | Motivation* | - Willingness to learn about online sales tools | Tailored to those who never checked groceries sales online (n=6) | 83.3 (5)  willing to learn* |
|  | Behavioral Skill | - Using digital methods to find grocery sales. - Using digital coupons | n= 196  Never  Every three months  Once or twice a month  Once a week  More than once a week  n= 196  Never  Every three months  Once or twice a month  Once a week  More than once a week | 41.8 (82)  1.0 (2)  18.4 (36)  22.4 (44)  16.3 (32)  54.6 (107)  1.0 (2)  15.3 (30)  15.8 (31)  13.3 (26) |
| **Online Grocery Use** | Information | - Recognize cost-saving delivery subscriptions | Discounted grocery delivery (n = 196)  No  I do not qualify  Yes  Don’t Know or Like to Know | 30.6 (60)  5.6 (11)  33.7 (66)  26.0 (51) |
|  | Motivation* | - Interest in online grocery shopping - Interest in digital tools to improve online grocery shopping experience | Tailored to those who never ordered groceries online (n = 90, 45.9%)  Tailored to those who haven’t downloaded a store app  (n = 37, 18.9 %)  Tailored to online grocery shoppers who haven’t use filters (n = 12, 6.1%)  Tailored to online grocery shoppers who haven’t added items from previous orders to the online grocery cart (n = 16, 8.2%) | 38.9 (35)  willing to order*  45.9 (17)  willing to download*  58.3 (7) willing to use filters*  50.0 (8) willing to use prefilled cart* |
|  | Behavioral Skill | - Online grocery ordering frequency - Use digital tools to improve online grocery shopping experience - Managing grocery costs | (n = 196)  Never  Every three months  Once or twice a month  Once a week  More than once a week  (n = 196)  Download a store app  Tailored to online grocery shoppers (n = 106, 54.1%)  Use filters to help find products  Add items from previous orders to the online grocery cart  Tailored to online grocery shoppers (n = 106, 54.1%)  Choosing curbside pickup at the grocery store  Picking the grocery store with the least amount of fees.  Memberships or subscriptions to lower delivery costs  Using digital coupons | 45.9 (90)  11.7 (23)  23.0 (45)  10.7 (21)  8.7 (17)  81.1 (159)  84.9 (90)  84.9 (90)  47.2 (50)  42.5 (45)  33.0 (35)  29.2 (31) |
|  | Confidence | - Perceive the ease of use of digital tools to improve the online grocery shopping experience | Tailored to online grocery shoppers (n = 106, 54.1%)  Buying food online is generally very simple  Filters help to find products  Saves time adding items from previous orders to the online grocery cart  Avoid buying certain foods (e.g., soda, and cookies)  Save money when ordering groceries online using digital coupons. | 70.8 (75)  68.9 (62)  77.8 (70)  20.8 (22)  62.2 (56) |
| **Digital Safety** | Information | - Recognize online scams | Tailored to online grocery shoppers (n = 106, 54.1%) | 60.4 (64) |
|  | Motivation* | - Willingness to learn about online safety | Tailored to individuals who have never ordered groceries online (n=90) and online grocery shoppers who did not correctly answer the safety practice question (n=42). Total (n=132, 67.3%) | 50.8 (67) |

*Participants were classified as motivated if they reported a score of ≥15 on the liking scale. The scale ranged from −100 to 100, with “love it”/“hate it” corresponding to ±75–100, “really like/dislike it” to ±45–75, “like/dislike it” to ±15–45, and “it is ok” to −15 to 15.

**Supplementary Material 4**

Food and Nutrition Literacy assessment items categorized by the Information-Motivation-Behavioral Skills (IMB) Model

| **Concepts** | **IMB Construct** | **Objectives** | **Survey Items** | **% (n)** |
| --- | --- | --- | --- | --- |
| **Healthy eating** | Information | **MyPlate**   - Recognize MyPlate. - Recall a MyPlate recommendation. | (n = 196)   - Remembering hearing about MyPlate.   Tailored to individuals who had heard of MyPlate (n = 149, 76%)   - Select that MyPlate recommends filling half the plate with fruits and vegetables. | 76.0 (149)  40.3 (60) |
|  | Motivation* | **MyPlate**   - Interest in learning about MyPlate.   **Healthy Eating Goals**   - Identify top three diet-related health goals.   **Nutrition Topics**   - Identify preferred learning topics related to eating healthy (e.g., meal planning, recipes). | Tailored to individuals who had heard of MyPlate (n = 149, 76%)  Select all that apply (n = 196)  To be healthier overall  Maintain a healthy weight  Increase my energy  Maintain a healthy body image  Feel better about myself  Help get my family on track with healthy eating  Get to a healthy weight  Reduce stress  Manage a health problem  None of the above  Select all that apply (n = 196)  Healthy eating on a budget  Recipes  Maintain a healthy eating lifestyle  Grocery shopping tips  Meal planning  Time-saving meal prep  Dining out/take out  Vegetarian meals | 58.1 (79)  willing to learn*  66.3 (130)  56.1 (110)  42.9 (84)  23.5 (46)  38.8 (76)  26.0 (51)  38.8 (76)  29.1 (57)  23.0 (45)  2.0 (4)  59.7 (117)  51.5 (101)  49.0 (96)  44.9 (88)  48.5 (95)  25.5 (50)  19.9 (39)  12.2 (24) |
|  | Behavioral Skill | **MyPlate**   - Uses online tools to learn more about and try MyPlate recommendations. | Tailored to individuals who had heard of MyPlate (n = 149, 76%)    Looking online for MyPlate  Trying MyPlate plan recommendations. | 39.6 (59)  59.7 (89) |
| **Nutrition Label** | Information | - Identifies barriers to using nutrition labels. - Using nutrition label to identify healthier product. | Tailored to individuals who responded No or Rarely to looking at the nutrition label (n=63, 32.2%)  Usually buy the same product  Takes too much time to read  Prefer getting information from other sources  Not interested in the nutrition information  More interested in the price of the product than the nutrition  Do not know  (n = 196)  Selected healthier food by comparing the nutrition label between two foods  Tailored to individuals who responded correctly (n=177, 90.3%)  Provided correct reason why the beverage is healthier. | 57.1 (36)  14.3 (9)  4.8 (3)  15.9 (10)  30.2 (19)  7.9 (5)  90.3 (177)  63.3 (124) |
|  | Motivation* | - Willingness to learn about reading labels. | Tailored to individuals who responded No, or I don’t know to looking at the nutrition label (n=40, 20.4%) | 52.5 (21) willing to learn* |
|  | Behavioral Skill | - Frequency of reading labels for dietary selection. | (n = 196)  No  Don’t know  Rarely  Once a month  Every time | 18.4 (36)  2.0 (4)  13.8 (27)  15.3 (30)  50.5 (99) |
|  |  | - Frequency of reading labels online | Tailored to online grocery shoppers and those who look at the nutrition label (n = 93, 47.4%)  Never  Very Rarely  Rarely  Occasionally  Frequently  Very Frequently | 9.7 (9)  6.5 (6)  6.5 (6)  20.4 (19)  35.5 (33)  21.5 (20) |
|  |  | - This six-question instrument measures whether individuals can use the nutrition facts label to calculate calories per serving, limit intake, deduct nutrients, and apply it to daily intake (4,5). | (n = 196)  **Score of 0-3** limited literacy  **Score of 4-6** adequate literacy | 41.3 (81)  58.7 (115) |
| **Unit Price** | Information | - Recognize unit price while shopping. | No  Don't know  Yes | 28.1 (55)  26.0 (51)  45.9 (90) |
|  | Motivation* | - Interest in learning how to use unit prices. | Tailored for individuals who do not use or don’t know about unit price (n = 106, 54.1%) | 58.5 (62)  Willing to learn* |
|  | Behavioral Skill | - Compares products using unit price. | Tailored for individuals who use unit price (n = 90, 45.9%)  Chooses better deal based on unit pricing | 66.7 (60) |
| **Food resource management** | Behavioral Skill | - The questionnaire measures food resource management through planning behaviors (such as identifying available food items, planning menus, and making a grocery shopping list) and saving behaviors (seeking sales or coupons, and comparing prices) (6) | (n = 196)  Frequency of looking in the refrigerator/ cupboard before shopping | Frequently + Very Frequently (Combined)  76.5 (150) |
|  |  |  | Frequency of planning meals before shopping for groceries. | 63.3 (124) |
|  |  |  | Frequency of preparing financially for food purchases. | 68.4 (134) |
|  |  |  | Frequency of making a list before shopping. | 65.8 (129) |
|  |  |  | Frequency of looking for sales before shopping. | 56.6 (111) |
|  |  |  | Frequency of looking for sales at the store. | 68.9 (135) |
|  |  |  | Frequency of using coupons for food savings. | 41.8 (82) |
|  |  |  | Frequency of engaging in price comparison to save money. | 71.5 (140) |

*Participants were classified as motivated if they reported a score of ≥15 on the liking scale. The scale ranged from −100 to 100, with “love it”/ “hate it” corresponding to ±75–100, “really like/dislike it” to ±45–75, “like/dislike it” to ±15–45, and “it is ok” to −15 to 15.

**Supplementary Material 5.**

Principal Axis Factoring (PAF) sensitivity analysis of the Digital Food and Nutrition Literacy (DFNL) index (n = 196).

**Sampling adequacy**

| **Statistic** | **Value** |
| --- | --- |
| Kaiser–Meyer–Olkin (KMO) | 0.695 |
| Bartlett's Test of Sphericity, χ²(df) | 424.50 (45) |
| p-value | <0.001 |
| Extraction method | Principal Axis Factoring |
| Rotation method | Varimax with Kaiser normalization |
| Number of factors retained* | 3 |

* To facilitate comparison with the primary PCA, the PAF sensitivity analysis specified extraction of three factors, corresponding to the three-component solution identified in the PCA.

**Rotated Factor Matrix**

| **DFNL indicator** | **Factor 1** | **Factor 2** | **Factor 3** | **Communality (h²)** |
| --- | --- | --- | --- | --- |
| Frequency checking online grocery sales | 0.887 | — | — | 0.836 |
| Frequency using digital coupons | 0.874 | — | — | 0.805 |
| Frequency ordering groceries online | 0.356 | — | — | 0.150 |
| Frequency using social media for nutrition | — | 0.758 | — | 0.628 |
| Number of social media apps used | — | 0.450 | — | 0.312 |
| Number of nutrition information topics searched online | — | 0.443 | — | 0.292 |
| MyPlate awareness | — | 0.311 | — | 0.129 |
| Number of devices to access the internet | — | — | 0.639 | 0.418 |
| Number of digital tools to communicate with health professionals | — | — | 0.332 | 0.225 |
| Nutrition label Knowledge | — | — | — | 0.082 |

*Values <0.30 are suppressed.*

**References**

1.	Colby S, Zhou W, Allison C, Mathews AE, Olfert MD, Morrell JS, Byrd-Bredbenner C, Greene G, Brown O, Kattelmann K, et al. Development and Validation of the Short Healthy Eating Index Survey with a College Population to Assess Dietary Quality and Intake. Nutrients 2020;12:2611.

2.	Dietary Guidelines for Americans, 2020-2025 [Internet]. 2020 [cited 2023 Jan 13]. Available from: https://www.dietaryguidelines.gov/sites/default/files/2020-12/Dietary_Guidelines_for_Americans_2020-2025.pdf

3.	Xu R, Blanchard BE, McCaffrey JM, Woolley S, Corso LML, Duffy VB. Food Liking-Based Diet Quality Indexes (DQI) Generated by Conceptual and Machine Learning Explained Variability in Cardiometabolic Risk Factors in Young Adults. Nutrients 2020;12:882.

4.	Weiss BD. Quick Assessment of Literacy in Primary Care: The Newest Vital Sign. The Annals of Family Medicine 2005;3:514–22.

5.	Grutzmacher S, Munger A, Messina L, Downes K. Screening for Health Literacy among SNAP-eligible Adults Using the Newest Vital Sign: Implications for Nutrition Facts Label Policy and Education. J Hunger Environ Nutr 2020;15:339–52.

6.	Barale K, Aragón MC, Yerxa K, Auld G, Hess A. Development of Reliable and Valid Questions to Assess Food Resource Management Behaviors in Adults With Limited Income. J Nutr Educ Behav 2022;54:346–58.
